# Association of Melioidosis Incidence with Rainfall and Humidity, Singapore, 2003–2012

**DOI:** 10.3201/eid2101.140042

**Published:** 2015-01

**Authors:** Xiang Liu, Long Pang, Siew Hoon Sim, Kee Tai Goh, Sharada Ravikumar, Mar Soe Win, Gladys Tan, Alex Richard Cook, Dale Fisher, Louis Yi Ann Chai

**Affiliations:** National University Health System University Medicine Cluster, Singapore (X. Liu, S. Ravikumar, M.S. Win, D. Fisher, L.Y.A. Chai);; National University of Singapore and National University Health System Saw Swee Hock School of Public Health, Singapore (L. Pang, K.T. Goh, A.R. Cook);; Defence Medical and Environmental Research Institute, Singapore (S.H. Sim, G. Tan);; Ministry of Health, Singapore (K.T. Goh);; National University of Singapore Yale-NUS College, Singapore (A.R. Cook);; National University of Singapore Yong Loo Lin School of Medicine, Singapore (D. Fisher)

**Keywords:** Burkholderia pseudomallei, climate, rainfall, humidity, temperature, melioidosis, Singapore, weather, urban, bacteria, soil, rural

## Abstract

Soil has been considered the natural reservoir for the bacterium *Burkholderia pseudomallei*, which causes melioidosis. We examined 550 melioidosis cases that occurred during a 10-year period in the highly urbanized city of Singapore, where soil exposure is rare, and found that rainfall and humidity levels were associated with disease incidence.

The gram-negative, saprophytic bacillus *Burkholderia pseudomallei*, which causes melioidosis, is endemic in northern Australia and Southeast Asia countries such as Thailand, Malaysia, and Singapore (*1*). Soil has traditionally been described as the natural reservoir of *B. pseudomallei* (hence the synonym “soil bacteria”) (*2*,*3*). Symptoms and signs of melioidosis can be mild, but severe manifestations such as bacteremia, organ abscesses, and severe pneumonia can lead to high death rates (*4*).

Two reports from northern Australia and northeastern Thailand, both conducted in predominantly rural areas, found an increase in melioidosis cases after heavy rainfall or extreme weather events such as tropical storms or monsoons (*5*,*6*). In Singapore, however, the occurrence of melioidosis in association with climatic variations has not been extensively studied. Geographically, Singapore has an urbanization rate of 100%, in contrast to northeastern Thailand (34%) and northern Australia (55%) (*7*,*8*). Because soil is the presumptive reservoir for *B. pseudomallei*, exposure may be less common in an extensively developed, urban setting such as Singapore, but other environmental factors, such as humidity and temperature, might also influence the pathogenicity of *B. pseudomallei*. We investigated 550 cases of melioidosis that occurred during a 10-year period in Singapore to determine if climatic factors might be related to the epidemiology of this disease in an urban setting.

## The Study

Melioidosis is a notifiable infectious disease in Singapore. Clinical and laboratory criteria for notification according to the Ministry of Health, Singapore, are a positive culture of *B. pseudomallei* (which accounts for 96.5% of notified cases) or strongly positive serologic test results combined with appropriate clinical manifestations (*9*). For our investigation, we obtained weekly melioidosis case numbers compiled on the basis of disease onset dates during 2003–2012 from the Ministry of Health, Singapore (*10*). Data on patient sex, age, and race were also included. Monthly and weekly rainfall, humidity, and temperature humidity data were obtained from the Singapore Meterological Service, Ministry of Environment and Water Resources, and from Weather Underground (http://www.wunderground.com). 

To assess the correlation between the incidence of melioidosis and rainfall, humidity, and temperature, we built regression models that used a quasi-Poisson distribution for the number of cases; Poisson and quasi-Poisson models are suitable for count data, of which the quasi-Poisson requires fewer assumptions. These models analyzed data at monthly and weekly intervals throughout the study period. Wald tests (i.e., the standard statistical test for regression models with nonnormal distributions, such as logistic and Poisson regressions) were conducted for various time lags after illness onset. Statistical significance was set at p<0.05. Statistical analyses were performed by using R Statistical Software version 3.0.1 (R Foundation for Statistical Computing, Vienna, Austria).

During the 10-year study period, 550 cases of melioidosis (range 31–96 cases per year) were notified in Singapore (Table 1). Of the patients, 84.1% were male, a higher percentage than found in previous studies in Thailand (57% [*11*]) and 69% for northern Australia (69% [*1*]). Mean patient age was 51.3 years. 

**Table 1:** Melioidosis case distribution by year, Singapore, 2003–2012*

Category	2003†	2004	2005	2006	2007	2008	2009	2010	2011	2012	Total cases
No. cases	44	96	74	59	57	60	37	58	34	31	550
Mean patient age, y	52.6	51.3	51.2	50.6	56.9	49.6	54.1	55	46.1	45.9	51.3
Patient sex											
F	6	15	9	13	7	9	8	13	6	1	87 (15.9)
M	36	81	65	46	50	51	29	45	28	30	461 (84.1)
Race/ethnicity											
Chinese	27	62	36	35	25	35	19	28	16	11	294 (65.6)
Malay	10	14	20	11	17	14	4	16	8	9	123 (27.5)
Indian	3	11	10	7	14	8	5	2	4	5	69 (15.4)
Others	0	4	2	0	0	3	7	3	4	0	23 (5.1)
Foreigners	2	5	6	6	1	0	2	9	2	6	39 (8.7)
Incidence by race											
Chinese	0.9	2.3	1.3	1.3	0.9	1.3	0.7	1	0.5	0.4	1.1
Malay	2	2.9	4.1	2.2	3.5	2.8	0.8	3.2	1.6	1.8	2.5
Indian	0.7	3.8	3.2	2.2	4.5	2.5	1.5	0.6	1.1	1.4	2.2
Total incidence	1	2.3	1.7	0.7	1.2	1.2	0.7	1.1	0.7	0.6	1.1
Deaths	6 (13.6)	26 (27.1)	12 (16.2)	9 (15.3)	12 (21.1)	12 (20.0)	5 (13.5)	14 (24.1)	6 (17.6)	2 (6.5)	104 (19.0)

*Values are no. (%) except as indicated. †Data for 2 cases in 2003 were unavailable.

The overall incidence of melioidosis in Singapore during the study period was 1.1 cases per 100,000 population. Disease incidence was highest among Malays and Indians (2.5 and 2.2 per 100,000 population, respectively). The mortality rate from the disease was 19.0%, similar to that for northern Australia (14%) (*4*), which likely reflects similar of health care provisions for the 2 cities; in contrast, the mortality rate for Thailand was 43% (*11*). 

During the study period, increased case numbers were generally observed during July–October and in January. The average total monthly rainfall for the period was 192.5 mm ± 121.6 mm (range 6.3–765.9 mm), and the average humidity and temperature were 83.7 mm ± 2.5% (range 77.3%–88.5%) and 27.7°C ± 0.7°C (range 26.3°C –29.2°C), respectively. 

The Figure shows a plot of the variations in total rainfall, average humidity, and average temperature by month along with corresponding numbers of melioidosis cases. We found a significant correlation between the number of melioidosis cases and the volume of rainfall in the 1-week period before disease onset, with a hazard ratio (HR) of 1.40 per 100 mm increase in rain (95% CI 1.03–1.90; p = 0.03) (Table 2). The humidity level 2 weeks before disease onset was more modestly associated with the number of cases (HR 1.03 per 1% increase in humidity, 95% CI 1.00 –1.05; p = 0.04), but this value did not have an independent association beyond that of rainfall in multivariable analysis; rainfall and humidity shared a positive correlation at a 1-week lag interval (R = 0.45; p<0.001). We found no association between temperature and the number of melioidosis cases.

**Figure:**
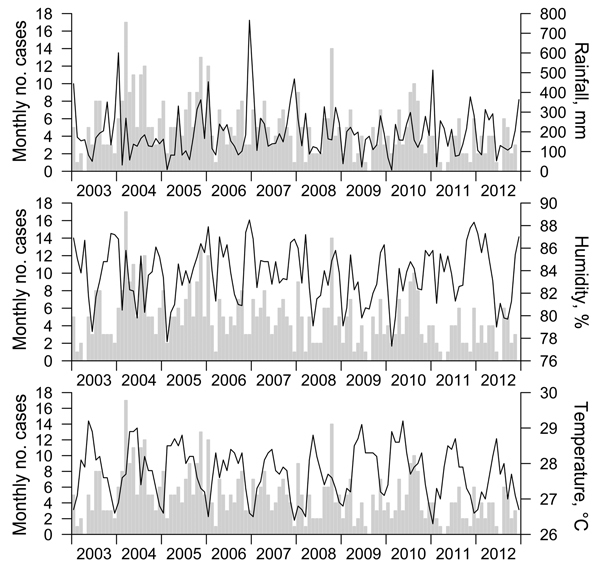
Total monthly rainfall (mm), average monthly humidity (%), and average monthly temperature (°C) compared with melioidosis case numbers, Singapore, 2003–2012. Cases are listed by date of illness onset.

**Table 2:** Temporal association of rainfall, humidity, and temperature with melioidosis cases, Singapore, 2003–2012*

Category	No lag			1-week lag			2-week lag	
	Hazard ratio (95% CI)	p value		Hazard ratio (95% CI)	p value		Hazard ratio (95% CI)	p value
Rainfall	1.20 (0.868–1.65)	0.274		1.40 (1.03–1.90)	0.0345		1.34 (0.981–1.83)	0.0667
Humidity	1.01 (0.985–1.04)	0.436		1.02 (0.997–1.05)	0.0806		1.03 (1.00–1.05)	0.0382
Temperature	1.01 (0.910–1.13)	0.814		0.949 (0.851–1.06)	0.351		0.928 (0.832–1.03)	0.178

*Association between melioidosis cases and weather conditions during the week of disease onset (no lag), 1 week before disease onset (1-week lag), and 2 weeks before disease onset (2-week lag). Significance is indicated by p<0.05.

## Conclusions

Soil is considered the natural reservoir of *B. pseudomallei* (*2*,*3*), but in the highly urbanized city of Singapore, the likelihood of soil exposure predisposing to infection by *B. pseudomallei* may reasonably be considered to be low. We found a significant correlation of melioidosis cases in Singapore with higher rainfall totals and, to a lesser degree, to higher humidity levels. This finding indicates that water, rather than soil, may be the central vehicle for transmission and acquisition of this disease. Epidemiologic data from rural Thailand and northern Australia (*5*,*6*) suggest that incremental volumes of rainfall result in raising the water table on land, which leads to accumulation of *B. pseudomallei* on surface soil, which becomes a reservoir for inhalation of aerosolized bacteria. However, most (82.0%) patients with melioidosis in Singapore did not report occupational or recreational exposure to soil (Communicable Diseases Division, Ministry of Health, Singapore, unpub. data).

We found a 1-week interval between periods of heavy rainfall and increased cases of melioidosis; a comparable study in Australia cited a 14-day lag (*5*). However, our results are supported by observations from a 6-month epidemiologic investigation conducted in 2004 that described a relationship between the incidence of melioidosis and cumulative rainfall amounts 7 days before onset of illness (*12*). Our findings strengthen support for a possible link between melioidosis transmission and water by demonstrating a strong association between melioidosis case numbers and rainfall amounts 1 week before disease onset and humidity levels 2 weeks before disease onset.

The variations in intervals between rainfall and disease manifestation that we found are within the estimated incubation period of 1–21 days for melioidosis. However, this finding may also be accounted for by the existence of variations in genome sizes, intraspecies diversity, and virulence in the *B. pseudomallei* strains from diverse geographic locations (e.g., Thailand, Vietnam, Singapore, Australia) (*13*). The association between water and melioidosis is further strengthened by findings from a recent epidemiologic case–control, interview-based survey of patients, which found that exposure to rain and water inhalation were among the risk factors for acquisition of disease (*14*). 

In summary, we found that, in Singapore, a highly urban area where contact with soil is rare, the numbers of melioidosis cases are associated with higher rainfall totals and higher humidity levels in the weeks preceding illness onset. This finding indicates that water, rather than soil, may be the central vehicle for transmission and acquisition of *B. pseudomallei* infection.
